# Recent advances in low oxidation state aluminium chemistry

**DOI:** 10.1039/d0sc02686g

**Published:** 2020-06-30

**Authors:** Katie Hobson, Claire J. Carmalt, Clare Bakewell

**Affiliations:** Department of Chemistry, University College London 20 Gordon Street London WC1H 0AJ UK c.bakewell@ucl.ac.uk

## Abstract

The synthesis and isolation of novel low oxidation state aluminium (Al) compounds has seen relatively slow progress over the 30 years since such species were first isolated. This is largely due to the significant challenges in isolating these thermodynamically unstable compounds. Despite challenges with isolation, their reactivity has been widely explored and they have been utilized in a wide range of processes including the activation of strong chemicals bonds, as ligands to transition metals and in the formation of heterobimetallic M–M compounds. As such, attempts to isolate novel low oxidation state Al compounds have continued in earnest and in the last few years huge advances have been made. In this review we highlight the remarkable recent developments in the low oxidation state chemistry of aluminium and discuss the variety of new reactions these compounds have made possible.

## Abstract 2

Documenting the synthesis and isolation of novel low oxidation state aluminium (Al) compounds, which until recently has seen relatively slow progress over the 30 years since such species were first isolated.

## Graphical abstract

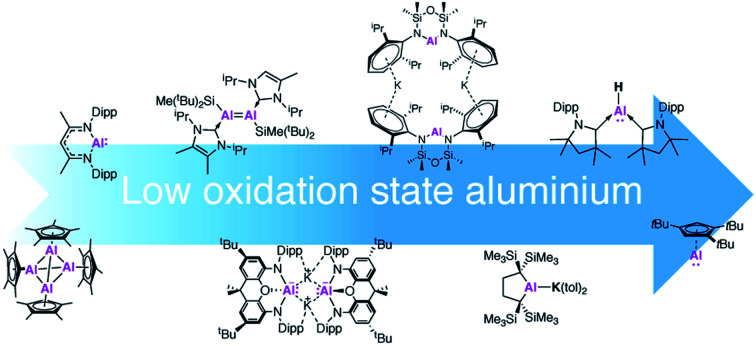

## Introduction

Recent years have seen a renaissance in main-group chemistry, with novel main-group compounds being shown to adopt unusual electronic configurations, demonstrate application in the activation of strong chemical bonds and facilitate a wide range of catalytic processes.^1–3^ In particular, the potential for main-group elements to adopt reactivity more commonly associated with transition metals is being widely explored, with redox type processes now becoming less chemical curiosities and more valuable synthetic methodologies.^4–7^ Aluminium species are one such class of main-group compound whose popularity has soared in recent years. As one of the most abundant elements in the Earth's crust, aluminium's low cost and low toxicity make it an attractive choice for a wide range of applications, from construction and electronics, to a key component in organic synthesis.^8,9^

The use of aluminium in major industrial catalytic processes dates back to the late 19^th^ century, when aluminium(iii) chloride was used to mediate the Friedel–Crafts reaction.^10^ In the 1950s Ziegler then demonstrated the use of aluminium hydrides in the Aufbau (chain growth) reaction of ethylene, a process that revolutionised modern manufacturing.^11^ In the ensuing years, aluminium's catalytic capabilities has been demonstrated in both hetero- and homogeneous systems for a range of applications spanning polymer chemistry, the synthesis of bulk commodity products and fine chemical production.^12–14^ Turnover in these homogenous catalytic cycles is typically controlled by the polarity induced between the Lewis acidic metal centre and the reactive metal substituent, Al^*δ*+^–X^*δ*−^, thus no change of oxidation at the metal is necessary. It was long believed that stable complexes of aluminium could only exist in the +3 oxidation state, owing to the thermodynamic instability of the +1 and +2 states. However, the late ‘80s’ and early ‘90s’ saw rapid developments towards the isolation of stable, molecular low oxidation state aluminium compounds, with general stabilisation strategies usually involving the use of sterically demanding and/or highly σ- and π-donating ligand systems. This includes work by Uhl and co-workers who reported the first example of an unsupported Al–Al bond [(R_2_Al-AlR_2_)] (where R = CH(SiMe_3_)_2_) and the low oxidation cluster K_2_[Al_12_^i^Bu_12_].^15,16^

**Fig. 1:**
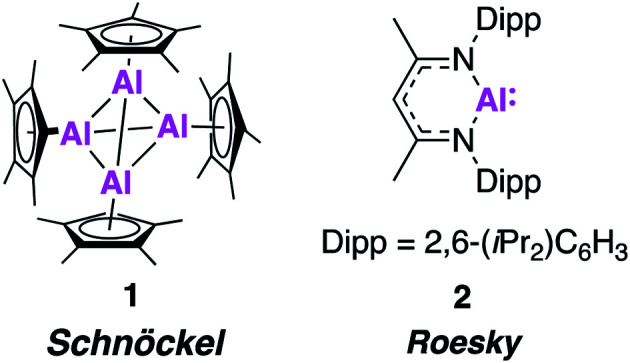
Structures of the first examples of stable aluminium (i) complexes.

In 1991, the first molecular Al(i) complex stable at room temperature was reported by Schnöckel and co-workers. The tetrameric complex [(Cp*Al)_4_] (**1**, Fig. 1) was synthesised by treatment of a metastable ethereal solution of AlCl with [Mg(Cp*)_2_], exploiting the steric bulk of the Cp* ligand (Cp* = pentamethylcyclopentadienyl). Further investigation has shown it can also be formed from the reduction of [Cp*AlCl_2_] and a redox reaction of [Cp*_2_AlH].^17–19^ Each Cp* ligand was *η*^5^-coordinated to the aluminium metal centre, which adopted a regular tetrahedral geometry. Compound **1** largely maintains its tetrameric structure in solution at room temperature, but can act as a masked source of Al(i) and has been shown to react with some small molecules, such as azides and oxygen, and deliver Cp*Al(i) as a ligand to transition metal centres.^20^ Its aggregated solution structure does however render it less potent a reducing agent than may be expected. In 2000, Roesky and co-workers made further progress in the advancement of low oxidation state Al chemistry, using the 2,6-diisopropylphenyl (Dipp) β-diketiminate ligand (BDI) to synthesise the first monomeric, solid state Al(i) complex (**2**, Fig. 1).^21^ Compound **2** was formed by the reduction of the parent diiodide complex, [BDIAlI_2_], with potassium metal and is structurally analogous to a carbene. Single crystal X-ray analysis revealed a well-defined monomer, with longer Al–N bond lengths and a more acute N–Al–N bond angle (1.957(2) Å, 89.86°) compared to the related Al(iii) dimethylaluminium compound (1.922 Å, 96.18(9)°). These properties are consistent with a change in oxidation state as the Al–N bond becomes less polar due to the reduced positivity of the metal centre. Compound **2** has been extensively investigated as a stoichiometric reducing agent for a range of challenging bond cleavage and small molecule activation reactions. Examples include the oxidative addition of H_2_ and C–F bonds across the Al(i) centre, as well as the reversible addition of alkenes and facilitation of CO homologation in the presence of a transition metal fragment.^22–25^ This reactivity has been comprehensively documented in several recent review articles.^5,20,26–28^ Despite the diverse range of reactivity demonstrated by Al(i), up until recently **1** and **2**, and a handful of derivatives thereof, have essentially remained the sole examples of stable sources of this powerful reagent. However, problems with their unreproducible and low yielding syntheses have hampered their use in more scalable synthetic procedures. For instance, **2** is typically formed in ∼20% yield, likely due to over reduction under the harsh reducing conditions. Attempts to make more synthetically attainable and ultimately more powerful sources of low oxidation state aluminium have therefore been pursued. The last few years have seen an explosion in this field, with the isolation and characterisation of a range of complexes that offer exciting new reactivity. In this review, we focus on these monumental advances that have set the stage for the future of main-group chemistry with commentary on the development of (1) aluminium–aluminium multiple bonds; (2) nucleophilic aluminium(i) alumanyl anions; (3) new monomeric aluminium(i) species and (4) the isolation of aluminium(i) hydrides.

### Aluminium–aluminium multiple bonds

In addition to the handful of isolated Al(i) species, numerous examples of Al–Al bonded compounds have been reported, some of which have bond orders greater than 1.^15,29–33^ There have also been examples of homodinuclear multiple bonds of group 13 elements including boron, gallium and indium.^34^ However until recently there were no known examples of a neutral complex containing a multiple bond between two aluminium centres, an important target if the metal–metal bond is to be partially retained during subsequent reaction.

**Fig. 2:**
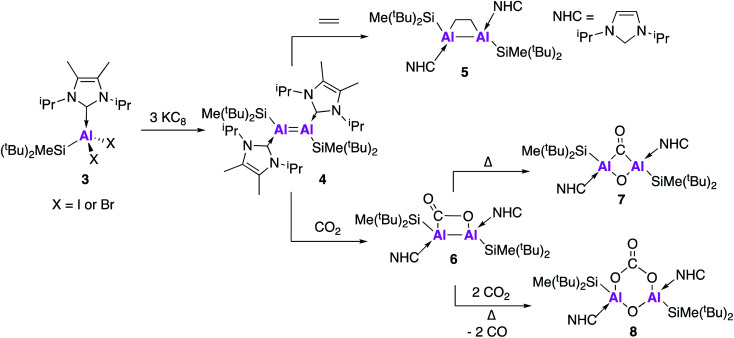
The synthesis of Al=Al doubly bonded compound **4** and its reactions with small molecules.

In 2017, Inoue and co-workers reported the first clean and high-yielding synthesis of a stable neutral compound containing an Al=Al double bond (**4**, Fig. 2).^35^ In a similar manner to **1**, compound **4** was synthesised by the reduction of an N-heterocyclic carbene (NHC) stabilised dihalide (**3**) with three equivalents of KC_8_. As will be shown through forthcoming examples, this use of metal halide precursors and strong reducing agents has become a common strategy to access low oxidation state aluminium centres. Single crystal X-ray diffraction (XRD) revealed a *trans*-planar geometry with an Al–Al bond length of 2.3943(6) Å. This is significantly shorter than previous examples of structurally characterised dialuminum compounds.^30,31,33^ Density functional theory (DFT) calculations showed that the highest occupied molecular orbital (HOMO) was the Al–Al π-bond and the HOMO−1 the Al–Al σ-bond. The Wiberg bond index (WBI), a bond order descriptor, was calculated to be 1.70, suggesting significant double bond character. A series of reactivity studies were conducted to prove the formation of an Al=Al double bond; firstly **4** was exposed to ethylene gas at room temperature from which a dialuminacyclobutane product was formed *via* a [2 + 2] cycloaddition process (**5**, Fig. 2). Compound **4** also reacted with phenylacetylene, this time forming two products, the [2 + 2] cycloaddition product and the terminal C–H insertion product. In all cases, the Al–Al bond was significantly longer than in the parent compound, with WBIs of approximately half, which support the presence of an Al=Al double bond in **4**. The reactivity of **4** has been further explored, with the stoichiometric activation of carbon dioxide (CO_2_) shown to be possible under mild conditions (Fig. 2).^36^ Exposure of **4** to 1 atm of CO_2_ resulted in the formation of **6** which is the formal [2 + 2] cycloaddition product from CO_2_ fixation, with single crystal XRD revealing a planar Al–Al–C–O ring. Heating **6** for an additional 60 minutes at 50 °C yielded **7**, which was found to contain a completely planar Al–(CO)(O)–Al core, comprised of a bridging carbonyl and oxo unit, representing the first example of a dialuminacarbonyl compound. **7** was calculated to have formed *via* the isomerisation of **6** in an overall exothermic process. The initial endergonic C–O cleavage step (+24.9 kcal mol^−1^) was offset by carbon monoxide (CO) recombination and the formation of a 4-membered ring (−42.4 kcal mol^−1^). Compound **8** was obtained by further heating of **6** under an atmosphere of CO_2_ and is comprised of a six-membered ring, with a bridging carbonate group and oxygen between the Al centres. The mechanism of formation of **8** was probed computationally and was found to be accessible *via* two different routes. The first route proceeded *via***6** through a series of six- and five-membered rings and was found to be exothermic (−77.3 kcal mol^−1^) and produce **8** as a single isomer. The second route sees **4** react with one equivalent of O_2_ to form a four-membered planar Al–(O)(O)–Al ring. This was found to be exothermic (−36.5 kcal mol^−1^) and proceeded *via* breaking of the 4-membered ring, thus allowing for recombination at either face of the Al-centre and a mixture of products to be obtained. This route could be borne out experimentally, leading to the formation of a 50 : 50 mixture of both the *cis* and *trans* isomers of **8**, suggesting the first route to be the most likely mechanism connecting **6** and **8**.

**Fig. 3:**
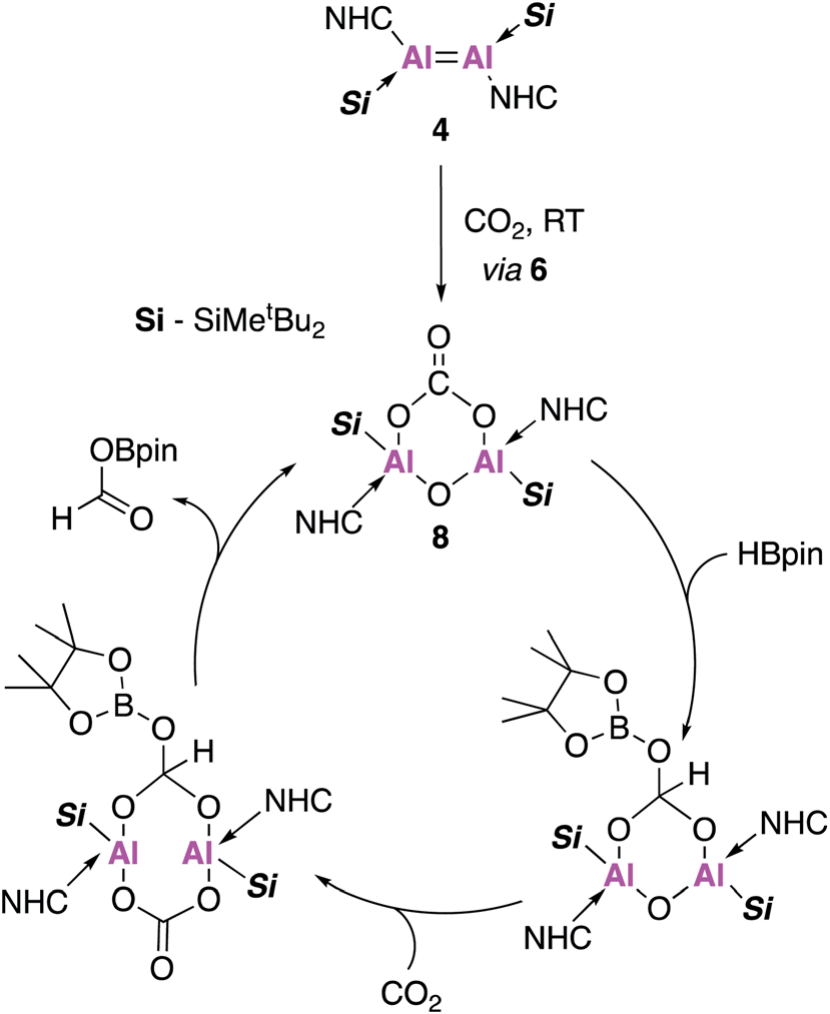
The proposed catalytic cycle for the hydroboration of CO_2_ using **4** as a pre-catalyst.

Compound **8**, formed *in situ* from **4**, was found to be active towards the catalytic reduction of CO_2_ using 4,4,5,5-tetramethyl-1,3,2-dioxaborolane (HBpin) as a substrate (Fig. 3). A combined mechanistic and computational study revealed an overall exothermic transformation (−13.4 kcal mol^−1^), proceeding *via* initial coordination of HBpin to the exocyclic oxygen of the carbonate fragment. An endergonic hydride transfer, found to be the rate determining step (+22.2 kcal mol^−1^) was proposed, which was energetically offset by the formation of the reduced carbonate species (−26.6 kcal mol^−1^). Finally, CO_2_ coordination to the opposite plane of Al⋯Al allowed for reformation of **8** and released the formic acid derivative as the CO_2_ reduction product (Fig. 3).

**Fig. 4:**
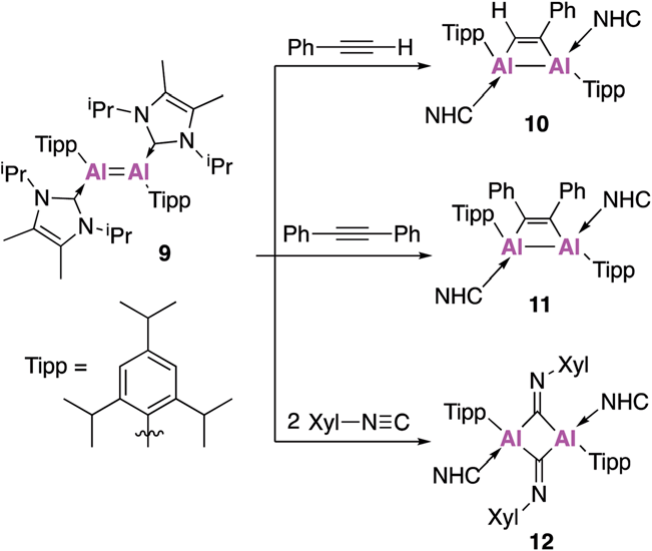
Reactivity of **9** (an analogue of **4**) with unsaturated molecules.

Further derivatisation of **4** was targeted in order to investigate the effects of different ligands on the geometry and reactivity of the Al=Al bond. Compound **9** (Fig. 4) was synthesised in an analogous fashion to **4**, with crystallographic analysis revealing a *trans*-bent and twisted geometry with nearly perpendicular arrangement of the NHC groups.^37^ This was notably different to **4**, whose NHC groups were orientated parallel to each other. The Al–Al bond length of 2.4039(8) Å was also slightly longer than in **4**. DFT analysis saw a reduced HOMO–LUMO gap between **9** (1.86 eV) and **4** (2.24 eV), with differences in the electronic structures attributed to the difference in steric demands of the stabilising ligands (silyl *vs.* aryl). Compound **9** reacts with ethylene in an analogous fashion to **4**, however reaction with phenylacetylene saw formation of only the [2 + 2] cycloaddition product (**10**). Interestingly, **10** decomposed in solution to form styrene, which is proposed to form *via* an intramolecular C–H insertion pathway. Enhanced reactivity of **9** was demonstrated by the facile reaction with diphenylacetylene at room temperature to generate the [2 + 2] cycloaddition product **11** (no reaction was seen with **4** at elevated temperature). This enhanced reactivity was attributed to the increased electrophilicity of **9** and flexibility of the Tipp ligand allowing for easier access of sterically demanding reagents to the Al=Al core. Similarly, two equivalents of 2,6-dimethylphenylisocyanide reacted with **9** to form the bridging XylC = N **12**, whilst the same reaction with **4** yielded an indistinct mixture of products.

**Fig. 5:**
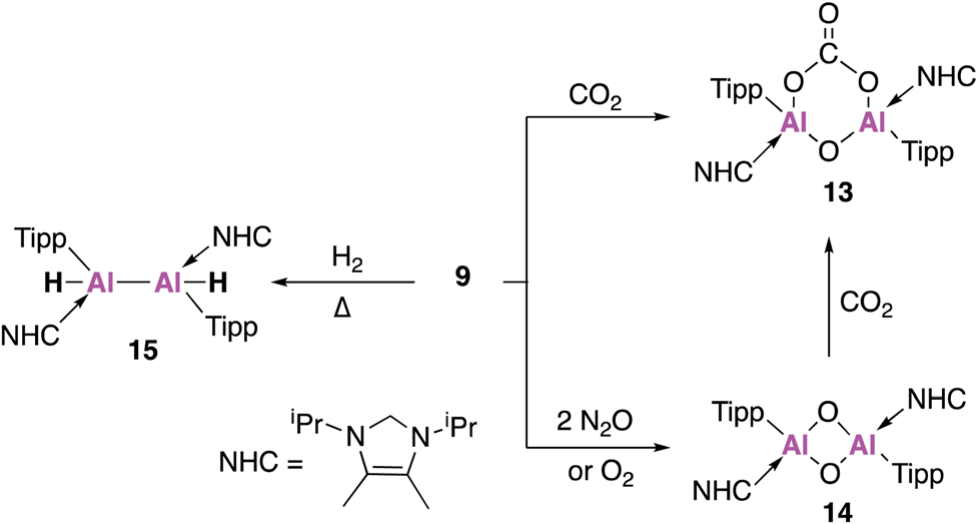
Reactions of **9** with small molecules.

Compound **9** underwent a [2 + 2] cycloaddition reaction with CO_2_ to form **13**, *via* a CO_2_ fixation product analogous to **6** (Fig. 5). However, the formation of both products could only be confirmed by the ^13^C NMR spectra due to the formation of numerous other compounds, attributed to the increased reactivity of **9**. The dioxo product **14** was obtained upon reaction of **9** with oxygen and, as was observed for **4**, further reaction with CO_2_ yielded carbonate complex **13**. Interestingly, compound **14** was not directly formed from the reaction of **9** with nitrous oxide (N_2_O) but instead formed a bridging aluminium(ii) mono-oxide species which, when further exposed to N_2_O, formed **14**. Reaction of **9** with dihydrogen (H_2_) was also explored; spectroscopic analysis indicated addition of H_2_ across the Al=Al double bond to form **15**. Single crystal XRD was not possible, but comparison of the observed IR frequencies with related terminal and bridging structures combined with DFT supported the formation of a structure with terminal hydrides and an Al–Al single bond. The activity of **9** was also tested in catalysis towards the hydroboration of CO_2_ and amine-borane dehydrogenation. The hydroboration of CO_2_ led to the facile formation of a number of reduction products, the ratio of which varied with respect to temperature. With **4**, only the singly reduced formic acid equivalent product was observed to form and this required long reaction times and high catalyst loading (Fig. 5). Amine borane dehydrogenation also saw an increased rate of conversion to a number of dehydrogenation products. The increased reactivity of **9** was attributed to its increased flexibility allowing greater access to the reactive core and nicely demonstrates how making simple changes to the ligand system can have a dramatic effect on reactivity. Further derivatisation of this class of Al–Al double bonded compounds has the potential to unlock even broader reactivity.

### Nucleophilic aluminium(i) alumanyl anions

**Fig. 6:**
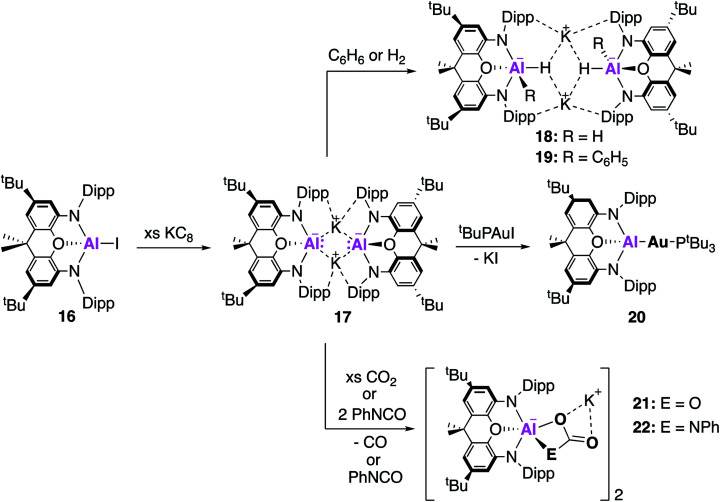
The synthesis and reactivity of the nucleophilic potassium alumanyl **17**.

Examples of charged low oxidation state aluminium complexes have also been the centre of much investigation in recent years. In 2018, the remarkable discovery by Aldridge, Goicoechea and co-workers of the first example of an anionic Al(i) nucleophile, [K{Al(NON)}]_2_, (**17**, NON = 4,5-bis(2,6-diisopropylanilido)-2,7-di-*tert*-butyl-9,9-dimethylxanthene, Fig. 6) was reported.^38^ This was synthesised by reduction of the aluminium iodide (**16**) with excess KC_8_ to obtain **17** as a centrosymmetric dimer. Compound **17** is comprised of two [Al(NON)]^−^ units and is stabilised by potassium–arene contacts. In an effort to rule out the presence of metal-bound hydrogens, [K{H_2_Al(NON)}]_2_ was synthesised, and all analysis supported the formation of two chemically distinct compounds. The Al–N bond distances in **17** (1.956(2) and 1.963(2) Å) were found to be longer than in **16** (1.846(2) Å), consistent with the formation of an Al(i) species. A series of reactions with electrophilic reagents was carried out, including H_2_ and benzene, resulting in the formation of multiple new aluminium-element bonds (Al–H, **18**; Al–C, **19**). This is in direct contrast to **2**, which only activates the strong C–H bonds of benzene in the presence of a palladium catalyst, highlighting its superior performance as a synthetic reagent.^39^ The highly donating alumanyl (sometimes referred to as aluminyl) complex was also employed as a ligand to stabilise electron rich transition metal compounds. Compound **17** reacted with one equivalent of [^*t*^Bu_3_PAuI] to form the two-coordinate gold complex **20**, which contained an Al–Au covalent bond.^40^ This structure was found to adopt a near linear geometry, with an Al–Au bond length of 2.402(3) Å. The effective atomic charges of the Al and Au centres were calculated to be +2.21 and −0.82, respectively, supporting the formation of a highly polarised Au^*δ*−^Al^*δ*+^ bond. Complex **20** therefore should react as a gold centred nucleophile, an assumption which was ratified by the reductive insertion of CO_2_ and diisopropylcarbodiimide into the Al–Au bond, forming products in which the gold centre was bound to the carbon.

The potassium alumanyl complex, **17**, could also be used to access an aluminium oxide ion, a compound that is of immense interest given the industrial use of aluminium oxide compounds in catalysis. The reaction of **17** with CO_2_ or phenyl isocyanate (PhNCO) gives the aluminium carbonate or carbamate products **21** and **22**, respectively (Fig. 6).^41^ Formation of both products was proposed to proceed *via* an aluminium oxide intermediate, though this could not be observed experimentally.

**Fig. 7:**
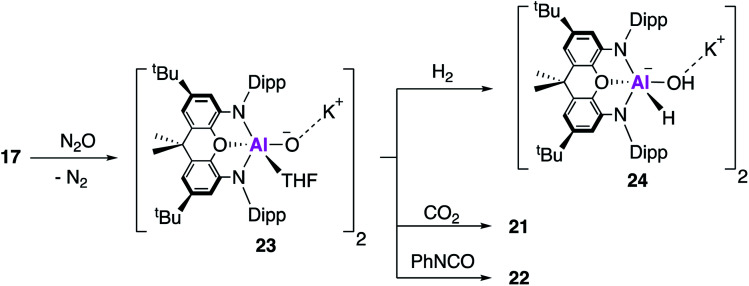
The formation of the aluminium oxide **23** and its subsequent reactivity with small molecules.

The aluminium oxide was therefore targeted directly; reaction of **17** with N_2_O at low temperature in tetrahydrofuran (THF) gives the THF ligated aluminium oxide **23** (Fig. 7). Single crystal XRD reveals a dimeric structure with two Al–O bond lengths measuring ∼1.68 Å, assigned as Al–O single bonds on the basis of DFT calculations which support the description of a highly polarised terminal bond. Compound **23** reacts with both CO_2_ and PhNCO at room temperature, to give the aforementioned products **21** and **22**, though no mechanistic investigation was conducted. Compound **23** was also able to react with H_2_ at room temperature and pressure, to give the aluminium hydride hydroxide **24**, the first isolated example of dihydrogen activation across an Al–O bond.

**Fig. 8:**
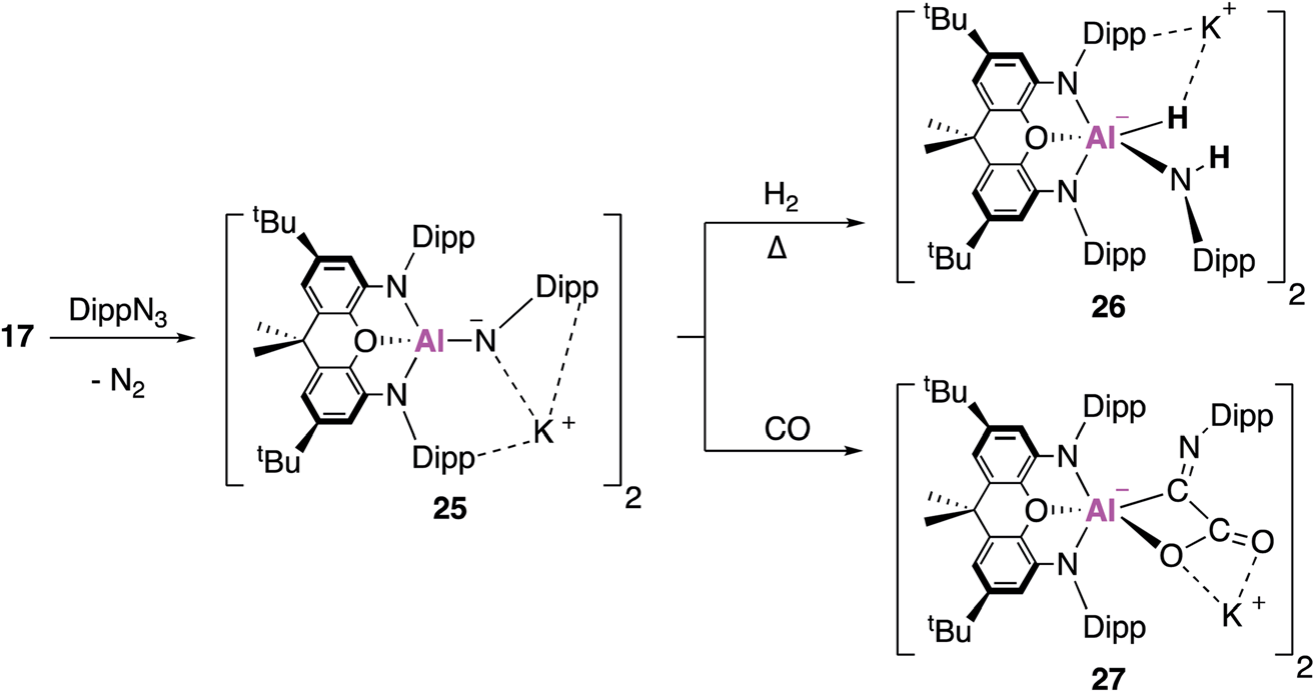
The formation of the aluminium imide **25** and its reactivity with CO_2_ and H_2_.

In related work, it was proposed that targeting the analogous imide complex, with a more shielded and less polarised Al–E bond, would lead to a greater degree of stability and reaction control than was offered by the aluminium oxide **23**. The imide complex, **25**, was synthesised from the reaction of **17** with the sterically hindered and less σ-donating 2,6-diisopropylphenylazide (DippN_3_) (Fig. 8); use of the smaller trimethylsilylazide led to the formation of the tetrazene complex or benzylic C–H activation of toluene.^42^ X-ray crystallographic analysis revealed **25** to be a centrosymmetric dimer, comprised of two anionic [(NON)Al(NDipp)]^−^ units bridged by K^+^ counterions, with Al–N bond lengths of 1.723(2) Å. Whilst the authors highlight this short bond length may suggest multiple bond character, they rule it out based on DFT which suggests the Al–N bond was best described as a highly polarised σ-bond. The propensity of **25** to activate small molecules was probed using dihydrogen and carbon monoxide. The reaction with H_2_ proceeded in an analogous fashion to **23**, but only after heating, to form the (amido)aluminium hydride **26**. Exposure of **25** to one atmosphere of carbon monoxide gave **27** as the sole product, formally derived from the assimilation of two CO molecules. The X-ray structure showed that the C–O bond lengths were consistent with the formation of a carboxylate bound through oxygen and the presence of a C=N double bond at the exocyclic carbon. This was proposed to proceed *via* carbon–oxygen triple bond cleavage and C–C bond formation; detailed DFT suggest an end-on approach of the first CO molecule to form a cyclic three-membered Al–N–C intermediate. The rate determining step was calculated to be isomerisation of this intermediate, forming a second intermediate where DippNCO was bound *via* the CO π-bond. CO bond cleavage led to a third intermediate, classed as the DippNC adduct of the analogous oxide [(NON)AlO]^−^, from which a barrierless assimilation of a further molecule of CO formed **27**.

**Fig. 9:**
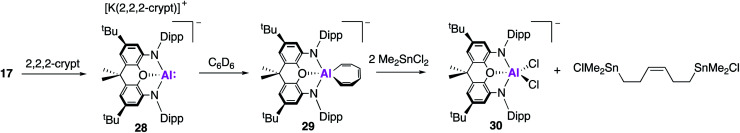
The formation of the monomeric alumanyl compound **28** and the C–C bond activation and functionalisation of benzene (**29–30**).

With the reactivity of **17** as a nucleophilic source of aluminium investigated extensively, a structurally analogous mononuclear species was targeted in order to study the effect of complex aggregation. The resultant monomeric species (**28**, Fig. 9) was synthesised *via* the addition of the K^+^ sequestering agent [2.2.2]cryptand to **17**.^43^ X-ray analysis revealed no short Al–Al or K–Al contacts whilst the NMR spectra exhibited characteristic differences compared to **17** and free [2.2.2]cryptand. Despite these differences, compounds **17** and **28** were both found to possess similar coordination geometries at the aluminium centre, consistent with the retention of an Al(i) species. Hence, **28** was best described as solution separated ions of both [K(2.2.2)crypt]^+^ and [(NON)Al]^−^. Assessment of the reactivity saw significant divergent reactivity between **17** and **28**. Reaction of **28** with benzene was found to proceed at room temperature and resulted in the activation of an aromatic C–C bond, to give **29**. This is in direct contrast to the observed reaction with **17**, which yielded **19***via* the formal oxidative cleavage of a benzene C–H bond (*vide supra*) and mirrors reactivity observed in photolysis reactions between 6-membered aromatic molecules and a dialkylsilylene.^44^ The resultant seven membered metallocycle, [AlC_6_H_6_] (**29**), had Al–C bond lengths consistent with a vinyl-aluminium species (1.980(2) Å), in conjunction with alternating double and single C–C bond lengths. The reversibility of C–C bond activation was probed by reacting complexes **28** and **29** with naphthalene. Both reactions yielded the 1- and 2- C–H activated naphthyl products in roughly equal ratios, supporting reversible C–C bond activation. Calculations revealed that the C–H activation of benzene was a significantly more exergonic process than C–C bond activation (−34.9 and −4.1 kJ mol^−1^), thus **29** forms as the kinetic product. Compound **29** was calculated to form from **28***via* a bicyclic intermediate from an initial [2 + 1] cycloaddition of benzene at the Al centre. The high energy HOMO of **28** is believed to facilitate the initial [2 + 1] cycloaddition step. In contrast, the same orbital in compound **17** was significantly stabilised by the dimeric K^+^-bridged [K(NON)Al]_2_ units, thus accounting for the observed pathways of reactivity. Expanding this reactivity further, it was proposed that this C–C bond cleavage could be exploited to form acyclic complexes *via* the reaction of benzene with electrophiles. The reaction of **29** with tin halides was shown to proceed at room temperature and yielded a di-tin species bridged by an acyclic C_6_H_6_ fragment, with retention of the benzene stereochemistry (Fig. 9).

**Fig. 10:**
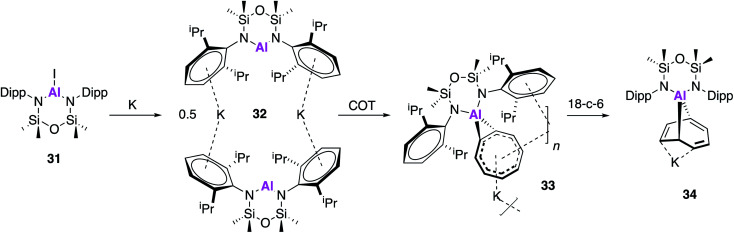
The synthesis and reactivity of the potassium alumanyl **32**.

Following on from the seminal isolation of NON–Al complexes, Coles and co-workers expanded this work by investigating the reactivity of a novel alumanyl anion with 1,3,5,7-cyclooctatetraene (COT).^45^ In an analogous fashion, the aluminium iodide (**31**) was reduced with potassium metal to form the alumanyl anion, **32**, which exists as dimer containing two anionic [Al(NON^Ar^)]^−^ units (Fig. 10). Unlike in **17**, the siloxane oxygen of the ligand backbone was found to be non-bonding, with Al⋯O distances of 3.356(2) and 3.418(2) Å and **32** can thus be assigned as a N-heterocyclic alumanyl anion. Reaction of **32** with COT led to the polymeric structure [K{Al(NON^Ar^)-(COT)}] (**33**), the first [Al–COT] complex with significant aromatic character. The Al–C bond lengths from the metal centre to the COT ligand (2.061(3) Å and 2.071(3) Å) were found to be longer than in other known Al(iii) compounds, confirming a *η*^2^-coordination mode. The crown ether, 18-crown-6, was added to a solution of **33** in an attempt to disrupt the polymer and as such the discrete [K(18-*c*-6)][Al(NON^Ar^)(COT)] (**34**) monomer was isolated. Interestingly, the coordination mode of the COT ligand changed to form the addition product 9-aluminabicyclo[4.2.1]nona-2,4,7-triene, which is reminiscent of the reactivity of **2** and related cyclic alkenes.^46^

**Fig. 11:**
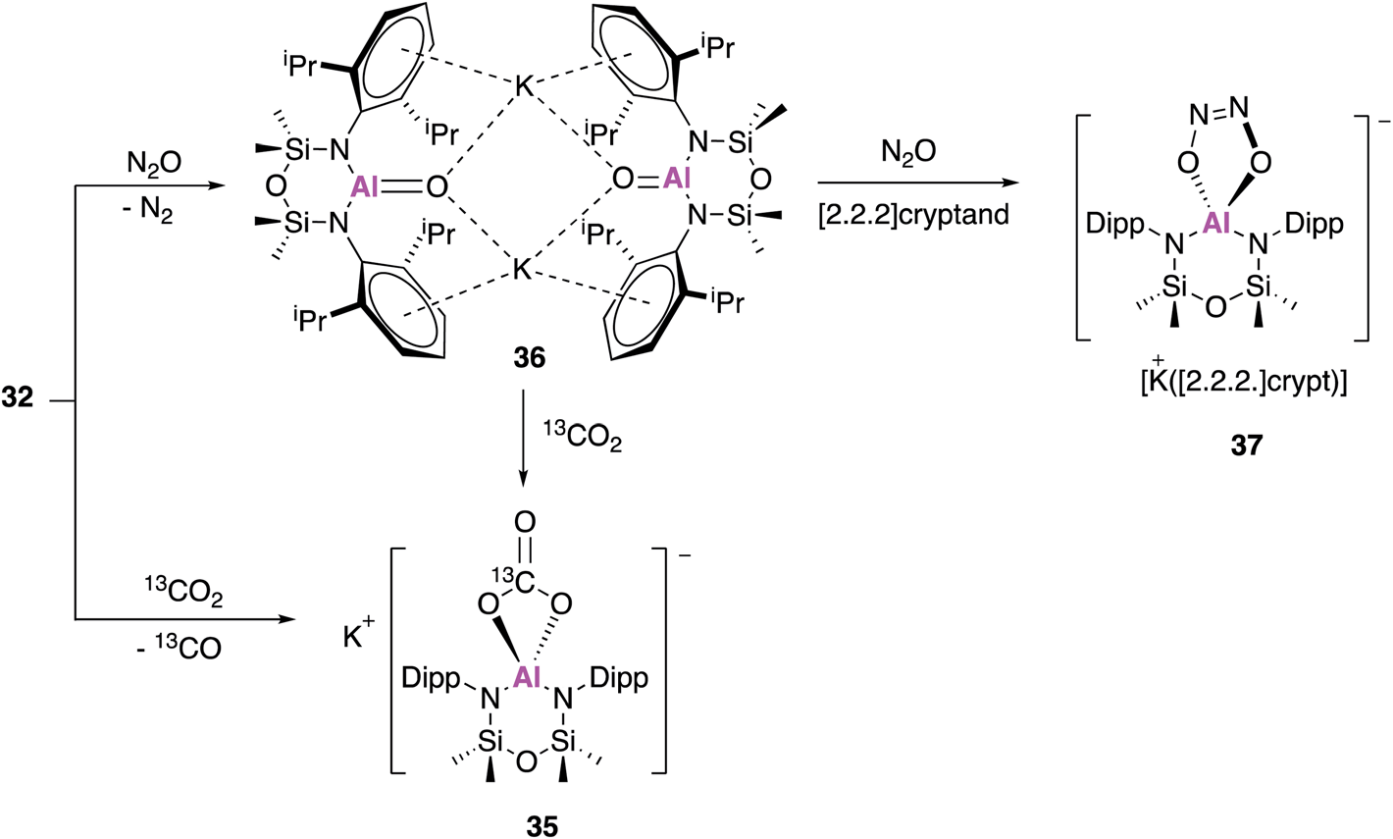
The reactivity of potassium alumanyl **32** with N_2_O and CO_2_.

In work published simultaneously to that of Aldridge and Goicoechea (**17**, Fig. 6–8), Coles and Anker sought to investigate the reactivity of **32** with CO_2_ and N_2_O. When **32** was exposed to one atmosphere of ^13^CO_2_ an analogous aluminium carbonate product **35** was observed to form *via* a [2 + 2] cycloaddition (Fig. 11).^47^ In an attempt to isolate the proposed aluminium oxide intermediate, **32** was also exposed to one atmosphere of N_2_O which resulted in formation of the dimer **36**, which comprised of [Al(NON^Ar^)(O)]^−^ anions and K^+^ counterions. The terminal Al–O bonds (1.6362(14) Å) were shorter than those of the related aluminium monoalumoxane species,^48^ and slightly shorter than those of compound **23**. The authors suggest this indicates multiple bond character, however both the calculated Wiberg bond index and NPA charges suggest ionic single bond character (note: **23** is assigned as an Al–O single bond). **36** reacts with ^13^CO_2_ to form **35**, supporting the hypothesis that a terminal Al–O bond is an intermediate in the formation of the carbonate species; this is analogous to reactivity observed with **23** to form **21**. Compound **36** was also found to react further with N_2_O in the presence of [2.2.2]cryptand to yield **37***via* a cycloaddition across the Al–O bond. These results suggest that the structurally analogous Al–O bonds of **23** and **36** may be described as having both single and multiple bond character.

**Fig. 12:**
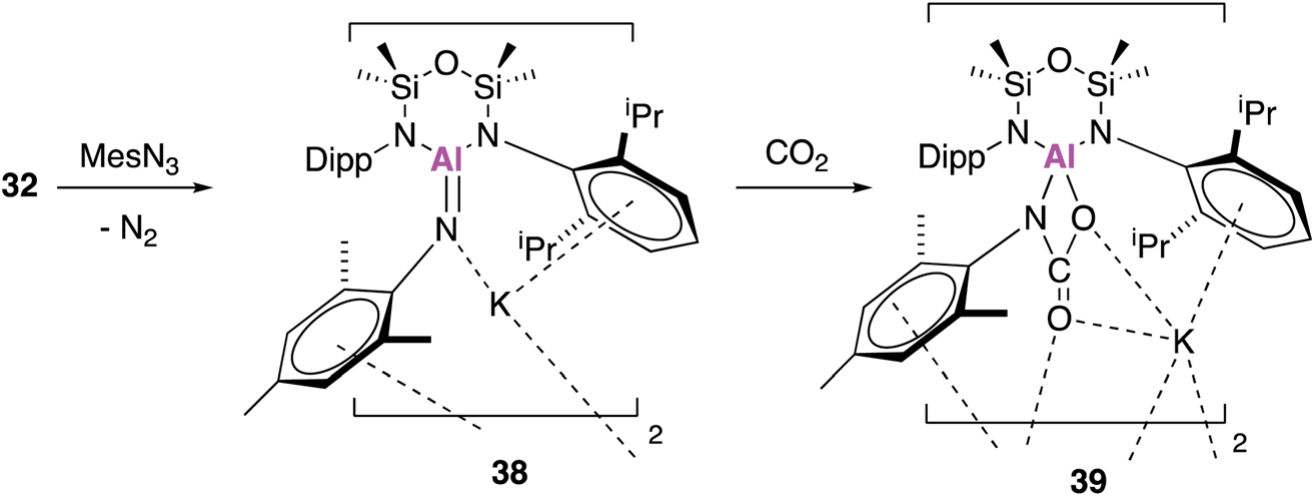
The reaction of potassium alumanyl **32** with mesityl azide.

Once again in simultaneously published research to **17**, **32** was reacted with mesityl azide, to yield an analogous three-coordinate aluminium complex containing a terminal aluminium-imide (**38**, Fig. 12).^49^ Again, single crystal XRD showed that **38** crystallised as a dimer, comprising of the aluminium imide salt K[Al(NON^Ar^)(NMes)] with an Al–N bond length of 1.7251(11) Å. This is comparable to the bond length in **25**, which was described as an Al–N single bond. DFT was used to probe the electronic structure of **38** and as with **25**, the WBI and NPA suggest a significant ionic component, however Kohn–Sham molecular orbitals indicate π-character. Therefore **38** (and **25**) may be better considered in resonance between Al^−^=N and Al–N^−^. With analogous reactivity to **36**, exposure of **38** to an atmosphere of CO_2_ saw complete conversion to the aluminium carbamate complex **39**, which was proposed to form *via* a [2 + 2] cycloaddition between the aluminium–nitrogen bond of the imide with a C=O of carbon dioxide.

**Fig. 13:**
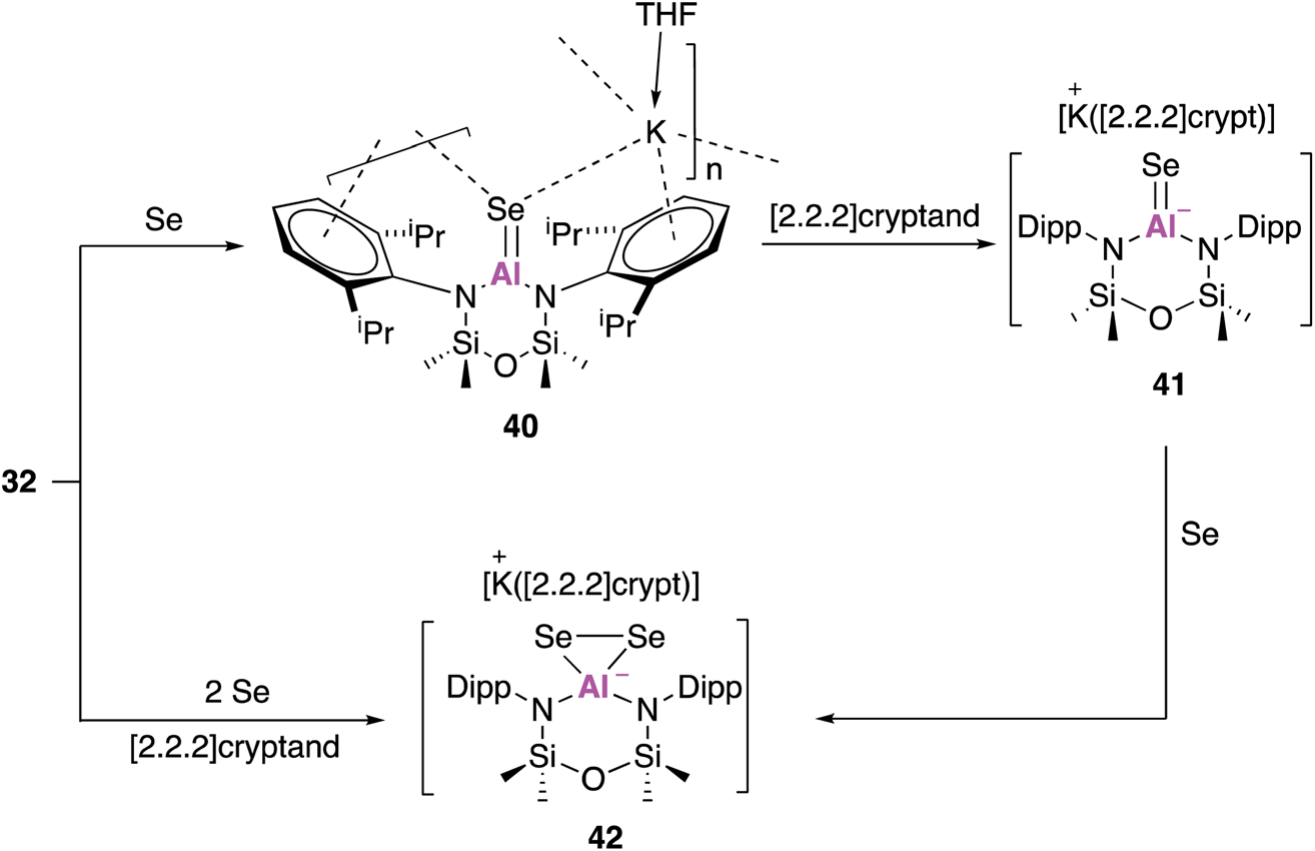
The reaction of potassium alumanyl **32** with selenium and related reactivity.

Compound **32** has also been used to access isoelectronic ‘heavy’ analogues of carbonyl functionalities.^50^ Reaction with one equivalent of selenium and catalytic amounts of tri-*n*-butylphosphine, as a selenium transfer agent, yielded the aluminium selenide (**40**, Fig. 13). Single crystal XRD revealed [Al(NON^Ar^)(Se)]^−^ anions linked to [K(THF)]^+^ cations through Se–K and π-arene interactions. The Al=Se bond length (2.2253(11) Å) was in good agreement with the sum of the molecular covalent double bond radii and shorter than terminal Al–SeH bonds. As with previous reactivity (Fig. 10 and 11), a sequestering agent ([2.2.2]cryptand) could be used break the coordination polymer and isolate Al=Se free from through space interactions. Compound **41** adopted a distorted trigonal planar geometry and a terminal selenium atom which exhibited the shortest Al–Se bond length ever reported (2.2032(6) Å). This was then used to target the selenium analogue of a dioxirane, by reaction with a further equivalent of selenium to afford **42**. Interestingly, **42** could also be obtained directly from the reaction of **32** with two equivalents of selenium and in the presence of [2.2.2]cryptand. The anionic components of **41** and **42** were probed computationally, and the calculated Al–Se bond lengths were found to be in good agreement with those observed experimentally. The Wiberg bond index of the Al–Se bond in **41** was calculated to be greater than that of an analogous Al–S bond indicating the presence of multiple bond character. This was further corroborated upon analysis of the orbitals whereby the HOMO was found to correspond to a π-symmetry lone pair on selenium, and the HOMO−1 contained lobes orthogonal to the HOMO extended across the Al–Se bond. The ability of this family of aluminium compounds to access such structures undoubtedly comes from the anionic nature of the aluminium fragment, allowing stabilisation of these isoelectronic heavy carbonyl species. Overall, investigations conducted with the alumanyl **32** emphasise the closely related reactivity with that of **17**, indicating that the differing ligand structure is having a negligible effect on the aluminium centre.

**Fig. 14:**
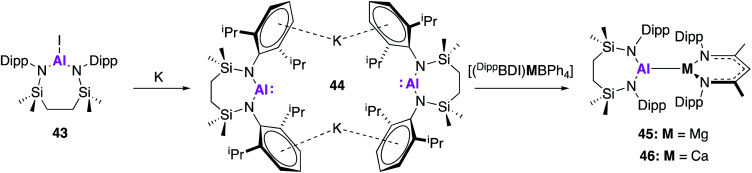
The synthesis of the potassium alumanyl **44** and subsequent formation of Al–M bonds.

In related work by Hill and co-workers, this class of nucleophilic aluminium reagents was expanded to include a seven-membered *N*,*N*′-heterocyclic potassium alumanyl species (**44**), which does not contain a bonding moiety in the ligand backbone.^51^ Compound **44** was synthesised analogously to **32**, *via* reduction of the aluminium iodide **43** over a potassium mirror (Fig. 14). Drawing inspiration from the reactivity of Roesky's [(^Dipp^BDI)Al] complex with related β-diketiminate complexes,^52–54^ the propensity of **44** to form metal–metal bonds with group 2 complexes was investigated. Alkaline earth alumanyls of the form [{SiN^Dipp^}Al–M(^Dipp^BDI)] (M = Mg, **45**; Ca, **46**) were synthesised upon addition of **44** to metal tetraphenylborate derivatives ([(^Dipp^BDI)MBPh_4_]; M = Mg or Ca). Single crystal XRD showed both complexes to comprise of an alkaline earth-aluminium bond, with each metal centre adopting a three-coordinate geometry. The Mg–Al bond length (2.798(6) Å) was longer than that of [(NON^Ar^)AlMg(^mes^BDI)] (2.696(1) Å), formed by reaction of **17** and (^mes^BDI)MgI(OEt_2_), but comparable to the Mg–Al bond in the previous reported compound [(^Dipp^BDI)Al(Me)Mg(^Dipp^BDI)].^52^ The Al–Ca bond in **46** (3.1664(4) Å) was longer than the sum of covalent radii for the metal centres, but was found to be in good agreement with a previously calculated structure for [(^Dipp^BDI)Al(H)Ca(^Dipp^BDI)] (3.12 Å).^53^ Complex **46** contains an additional *η*^6^ interaction between the calcium and the aryl substituent of the aluminium-coordinated diamide ligand which were not observed in **45**. DFT calculations were used to probe each structure further, and Pipek–Mezey orbital calculations revealed an intrinsic polarisation to both Mg–Al and Ca–Al σ-bonds. Natural bond orbital (NBO) calculations showed the Al–Ca interaction of **46** was slightly more ionic than the corresponding Mg–Al bond. Initial investigations into the reactivity of complexes **45** and **46** revealed that neither species reacted with aromatic solvents. Compound **45** remained stable whilst suspended in ethereal solvents but addition of THF to **46** in methylcyclohexane yielded a charge separated species comprised of [(^Dipp^BDI)Ca(THF)_3_]^+^ cations and [(SiN^Dipp^)AlO(CH_2_)_4_]^−^ anions. This was formally described as the oxidative addition product of a THF C–O bond to the Al(i) centre. Similarly, reaction of **46** with 1,3,5,7-cyclooctatetraene (COT) resulted in the two-electron aromatisation of the COT species and formed a heterobimetallic inverse sandwich species, whilst **45** was found to be unreactive. As the reactivity of **44** with small molecules such as H_2_ and CO_2_ has, to date, not been reported, it is difficult to draw conclusions on the effect of ligand variation compared to **17** and **32**. However, similarities in Al–Mg bond formation suggest parallels in reactivity are to be expected.

**Fig. 15:**
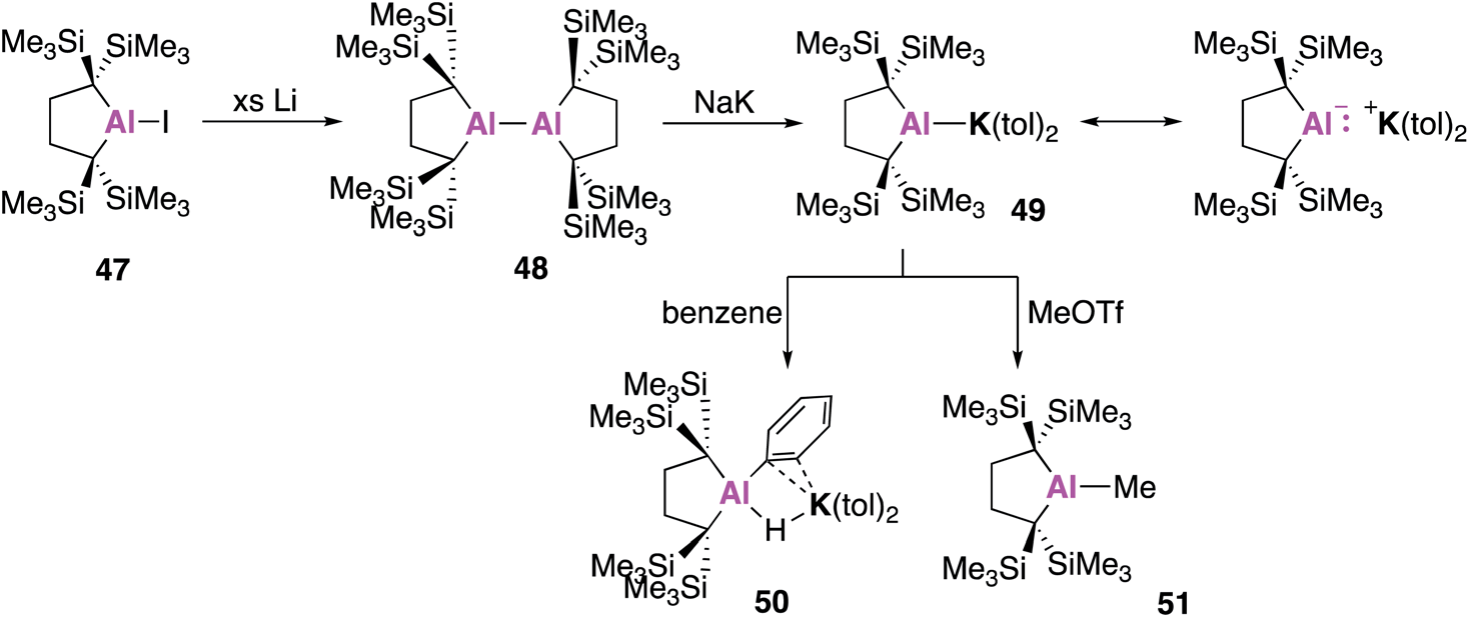
The synthesis of potassium alumanyl **49** and reactivity with benzene and MeOTf.

**Fig. 16:**
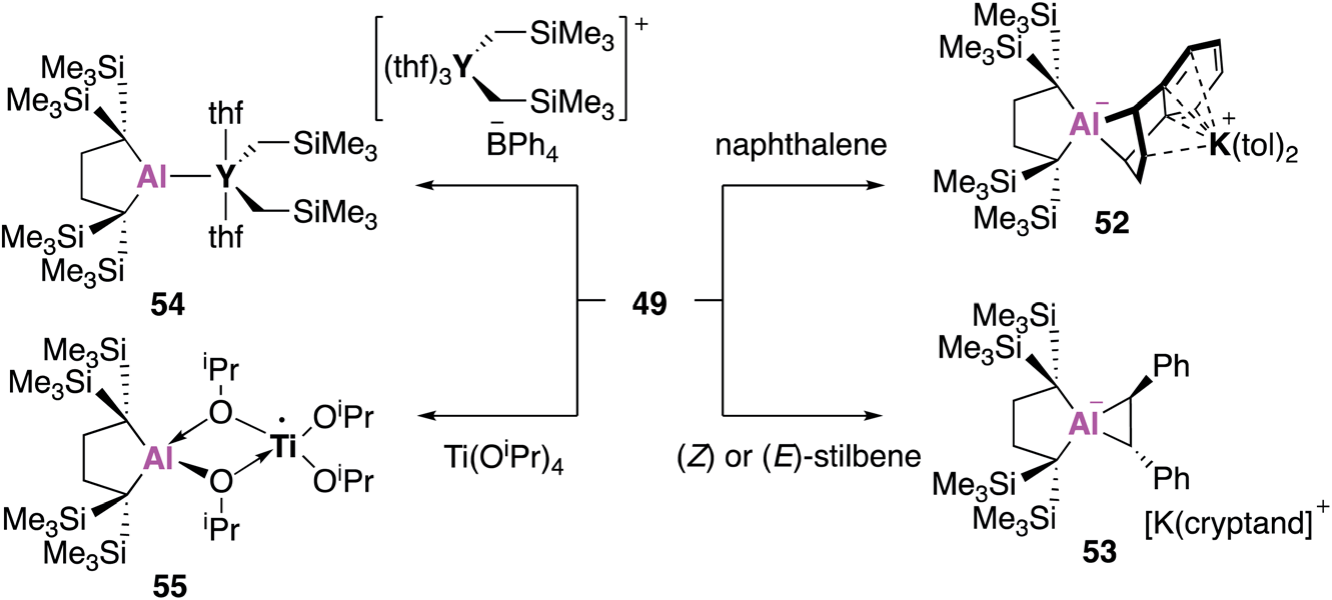
The reactivity of potassium alumanyl **49**.

All examples of base stabilised nucleophilic aluminium species discussed thus far contain nitrogen ligand systems, that offer additional stabilisation to the metal centre. Yamashita and co-workers proposed to explore the possibility of creating an ‘electronically unbiased’ group 13 anion, rationalising the tetrakis(trimethylsilyl)butylene ligand system to be an appropriate choice given its successful implementation in silylene chemistry.^55,56^ The corresponding aluminium iodide **47** could be synthesised in a two-step process from 1,1-bis(trimethylsilyl)ethylene, firstly by lithiation to form an etherate of 1,4-dilithio-1,1,4,4-tetrakis(trimethylsilyl)butane, followed by a salt metathesis with aluminium triiodide. Compound **47** was reacted with excess lithium to form the dialumane **48**, with subsequent treatment with sodium–potassium alloy (NaK) resulting in the formation of the potassium alumanyl **49** (Fig. 15). Single crystal XRD found **49** to exist as a monomeric Al–K complex, in contrast to the dimeric structures previously reported (**17**, **32**, **44**). The Al–K bond of length (3.4549(5) Å) was slightly longer than the sum of the covalent radii, 3.28 Å, and much shorter than observed in **17**, **32** and **44**.^38,45,51^ The Al–C bond lengths in **49** were longer than in **48** and the C–Al–C bond angle was wider, indicating a less electropositive Al centre. DFT analysis revealed the HOMO to be the lone pair of electrons centred mainly on the aluminium and the lowest unoccupied molecular orbital (LUMO) to be comprised of the π* orbitals of the potassium coordinated toluene molecules. The vacant 3p orbital of Al was found in the LUMO+8. The HOMO was higher in energy and the HOMO–LUMO gap was found to be significantly smaller than in **17** and **32**. This was proposed to be due to the absence of electron withdrawing nitrogen atoms and the associated Al–N pπ–pπ interactions in addition to the presence of the low-lying π* toluene orbitals. Both natural population analysis (NPA) and atoms in molecules (AIM) analysis show the Al–K bond in **49** can be considered as highly polarized. Compound **49** was found to deprotonate benzene under mild conditions (2.5 h, r.t.) to give the C–H oxidative addition product **50**, significantly faster than the related reaction between **17** and benzene (60 °C, 4 days). Reaction with methyl triflate lead to compound **51**, demonstrating the nucleophilicity of the aluminium centre. Interestingly, **49** was also found to activate C–F bonds in hexafluorobenzene, undergo nucleophilic substitution with the chloride atom in benzyl chloride and to facilitate [1 + 4] and [1 + 2] cycloaddition reactions with unsaturated hydrocarbons (Fig. 16).^57^ For instance, reaction of **49** and naphthalene gave the 1 + 4 cycloaddition product, **52**. Reaction of **49** with (*E*)- or (*Z*) stilbene gave the same *trans*-diphenylaluminacyclopropane [1 + 2] cycloaddition product **53**, with the *trans*-selectivity being confirmed by both single crystal XRD and ^1^H NMR analysis. DFT shows (*E*)-stilbene to react directly *via* a concerted pathway, but reaction of (*Z*)-stilbene proceeds *via* the formation of carbanionic intermediate containing a single C–C bond. This facilitates rotation around the C–C bond leading to the more thermodynamically favourable *trans*-product.

Reaction of **49** could also be extended to the formation of unsupported metal–metal bonds.^58^ Reaction of **49** with [Y(CH_2_SiMe_3_)_2_(THF)_3_][BPh_4_] led to the formation of **54**, the first example of a single aluminium–yttrium bond. The aluminium–yttrium bond length was found to be 3.1870(8) Å and the aluminium centre exhibited trigonal planar geometry. DFT was used to determine the electronic structure of **54**, with the HOMO representing the 2c–2e Al^−^–Y^+^ bond and the LUMO overlapping vacant 3p and 3d-orbitals. AIM calculations reveal a stronger Al–M bond in **54** than in **49**. In further reactions with metal precursors, **49** was also recently found to reduce titanium tetraisopropoxide to the trivalent titanium complex **55**.^59^ Overall, **49** has been shown to have enhanced reactivity towards molecules such as benzene than the dimeric alumanyl compound **17** (reactivity of **32** and **44** not reported) and a broad reaction scope. The use of the tetrakis(trimethylsilyl)butylene ligand system has led to a monomeric structure with a reduced HOMO–LUMO gap, leading to this heightened reactivity. However, recent work by Harder and co-workers has shown an anionic potassium alumanyl, formed *via* deprotonation of the BDI ligand in **2** with KN(SiMe_3_)_2_, to be capable of benzene C–H activation.^60^ Here, the potassium anion was found to stabilise the transition state, thus the role of potassium and possibility of bimetallic mechanism should be explored across this family of compounds.

**Fig. 17:**
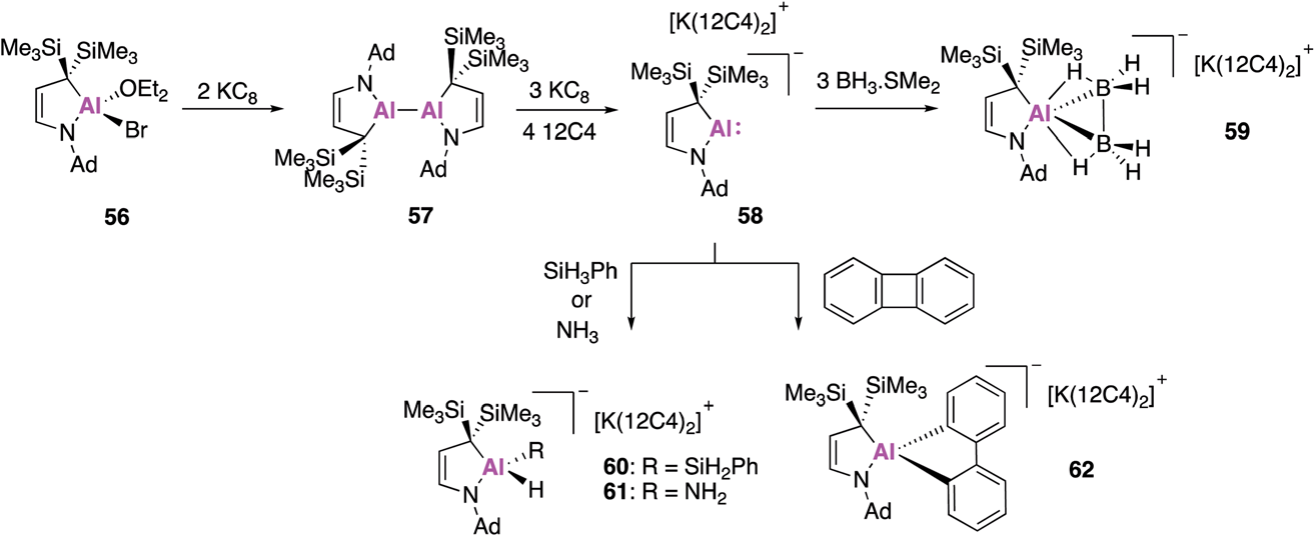
The formation of alumanyl **58** and its reactivity with small molecules.

Even further extension of the structural diversity of Al(i) anions was subsequently reported by Kinjo and Koshino, who targeted an aluminium analogue of a cyclic (alkyl)(amino)carbene (CAAC). CAACs are known to be more σ-donating and π-accepting than conventional NHCs and thus have the potential to have divergent activity from the aforementioned *N*,*N*-stabilised alumanyls (**17**, **32**, **44**) and the carbene analogue **49**. In a similar fashion, the formation of the cyclic (alkyl)(amino)alumanyl anion (CAAAl) **58** was achieved by reduction of the aluminium bromide **56** with potassium graphite in an apolar solvent, which yielded the dialane compound **57** (Fig. 17).^61^ This was followed by addition of diethyl ether and three further equivalents of potassium graphite, followed by the addition of 12-crown-4 ether to give the alumanyl anion product **58**. Compound **58** exists as a separated ion pair, similar to **28**, with sp^3^ hybridised C and N atoms bound to the Al centre. DFT of the aluminium anion revealed the HOMO to be the lone-pair at Al and the empty p-orbital to be the LUMO+1. The HOMO was found to be higher in energy than that of compounds **28** and **49**, though the HOMO–LUMO gap was larger. The reactivity of **58** was tested towards BH_3_·SMe_2_ and strong σ-bonds, the former leading to the formation of **59**, which contains a trigonal AlB_2_ ring. This type of reactivity has never before been observed in the main-group and **59** is the first isolated example of an aluminium complex of diborane. Reaction of **58** with phenylsilane or ammonia led to oxidative addition of an E–H bond to the Al centre, leading to **60** and **61** respectively. Formation of **60** represents the first example of a hydridic Si–H activation by an anionic aluminium species, though related reactions are known with the neural compound, **2**.^22^ Oxidative reactivity could also be extended to apolar σ-bonds and **58** was found to effect the C–C bond activation of biphenylene to form **62**. This diverse reactivity indicates **58** has the potential to access a wide range of aluminium bound molecular fragments that may be used in further synthesis, although the substrate scope investigated to date does not allow for direct comparison with other alumanyl species.

### New monomeric aluminium(i) complexes

Whilst huge advances have been made in the development of alumanyl anions, the isolation of new examples of monomeric aluminium(i) compounds remains an attractive target, especially considering the broad and well-defined reactivity displayed by Roesky's β-diketiminate complex (**2**). As mentioned previously, Schnöckel's Al(i) complex (**1**) is known to exist as a tetramer both in the solid state and in solution at room temperature, though an equilibrium with the monomeric form can be achieved by varying reaction temperature. However, differing substituents of the Cp ring allows for perturbation of this equilibrium which is dependent on both sterics and temperature.^62,63^ The tetrameric structure can hamper the reactivity of **1**, and over the years its use in small molecule activation has not been as widely explored as for **2**, whose discrete solid and solution state structure and ready solubility in common organic solvents renders it more user friendly.

**Fig. 18:**
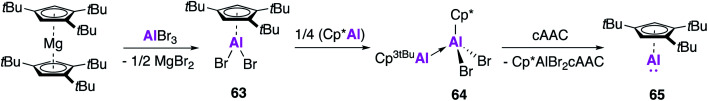
The synthesis of monomeric Al(i) species **65**.

Attempts to isolate monomeric forms of **1** by changing the sterics of the Cp ligand framework had not previously led to an easily isolatable monomeric Al(i) species. Accordingly, Braunschweig and co-workers targeted the formation of a monomeric Al(i) species that could be readily synthesised and isolated.^64^ The sterically demanding 1,3,5-tri-*tert*-butylcyclopenta-dienyl (Cp^3*t*Bu^) ligand was employed, and reaction of (Cp^3*t*Bu^)_2_Mg with AlBr_3_ saw the formation of the aluminium dibromide (Cp^3*t*Bu^)AlBr_2_ (**63**, Fig. 18). Compound **63** was shown to be a monomer in the solid state, where as the Cp* analogue is dimeric. The low coordinate Al(i) species **65** was then isolated first by a conproportionation reaction with (Cp*Al)_4_ to form **64**, followed by addition of a strong Lewis base (CAAC), after traditional reduction routes proved unsuccessful. The ^27^Al NMR spectrum showed a resonance at −161 ppm, indicative of monomeric Al(i) with no evidence of the tetramer and whilst formation of single crystals was not possible the ^1^H DOSY spectra also pointed towards a monomeric solution structure.

**Fig. 19:**
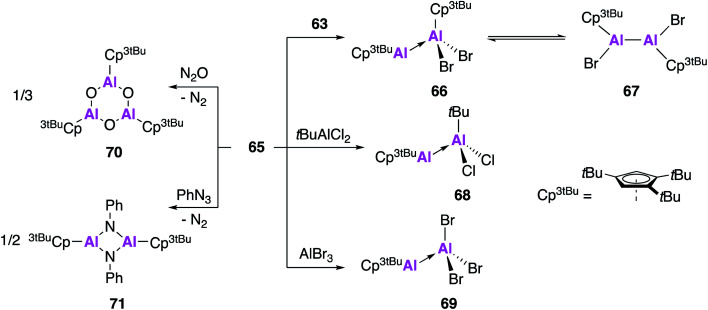
The reactivity of monomeric Al(i) species **65** with aluminium precursors and small molecules.

The reactivity of **65** was investigated with a number of aluminium precursors and small molecules (Fig. 19). Analysis of the reaction of **65** with **63** indicates the presence of valence tautomerism between the Al(i)–Al(iii) product **66** and the Al(ii)–Al(ii) product **67**, which appear to exist in rapid equilibrium but can be selectively isolated at different temperatures. Conversely, reaction with *t*BuAlCl_2_ or AlBr_3_ only led to formation of the Al(i)–Al(iii) Lewis acid–base adducts **68** and **69**, respectively. In an effort to target a monomeric Al–O containing complex, **65** was oxygenated with N_2_O, however what instead formed was the 6-membered heterocycle **70**, the formal oligomerisation product of Cp^3*t*Bu^Al=O. Further reaction of **65** with phenyl azide led to the formation of the imidoalane **71**, which was found to be dimeric in the solid state but showed evidence of monomeric solution behaviour. These reactions all proceed faster and often with more control than analogous reactions with (Cp*Al)_4_, highlighting the benefits of using the monomeric Cp-based Al(i) in synthesis.

**Fig. 20:**
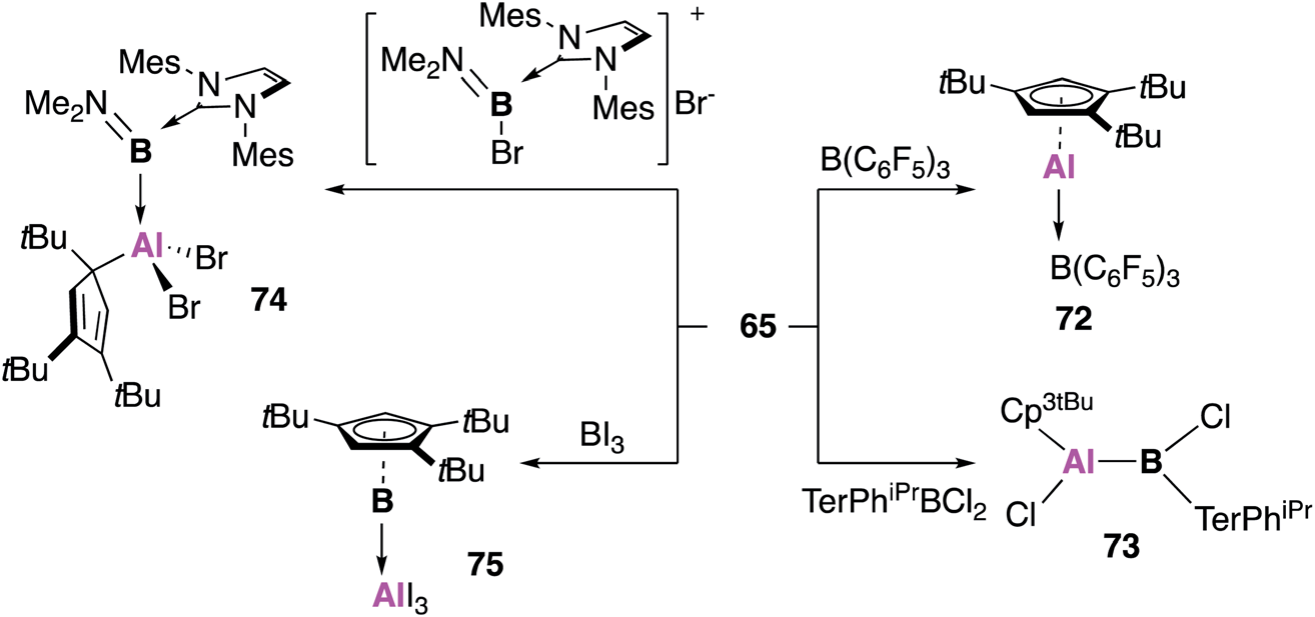
The reactivity of monomeric Al(i) **65** with boron(iii) species.

Braunschweig and co-workers further extended the reactivity of **65**, targeting compounds with direct unsupported aluminium–boron bonds (Fig. 20).^65^ Reaction of **65** with B(C_6_F_5_)_3_ also led to the formation of the acid–base adduct **72**, for which the analogous product was observed with both (Cp*Al)_4_ (**1**) and **2**.^66,67^ However, reaction with the other boron sources led to significantly divergent behaviour. The B–Cl bond of bis-2,6-(2,4,6-triisopropylphenyl)-phenyl dichloroborane was oxidatively added to **65**, leading to the covalent bora–alane complex **73**, which is possibly favoured over the adduct due to steric congestion at boron. The utility of **65** as a reducing agent was also demonstrated, with the redox formation of borylene–alane complexes. The reaction of **65** with the NHC stabilised boron species [(IMes)B(Br)NMe_2_]Br yielded the oxidative addition product **74**, which forms with oxidation of aluminium and reduction of boron (Al(iii)–B(i)). A redox reaction also occurred in the reaction of **65** with BI_3_, this time resulting from the rearrangement of substituents, leading to an AlI_3_ borylene adduct (**75**). This range of reactivity with boron underlines the highly versatile nature of **65**, and the benefits of using a monomeric source of CpAl(i).

### The isolation of aluminium(i) hydrides

Whilst examples of some classes of Al(i) species are slowly becoming more established, the Al(i) hydride ‘aluminene’ has remained more elusive. Al(i)–H has previously been studied in a matrix, but attempts to isolate it synthetically have proven challenging.^68^ In 2018, Jones and co-workers reported the isolation of an Al_6_H_6_ cluster, formed through the reaction of a magnesium(i) dimer and an aluminium(iii) dihydride.^69^ This was characterised in the solid state as a [Al_6_H_6_(HC(NDipp)_2_)_2_]^2−^ dianion stabilised by two [BDIMg]^+^ cations and is the first example of a stable Al(i)–H containing complex. A lack of solubility in common hydrocarbons precluded solution state characterisation and to date further reactivity has not been reported. Driess and co-workers have also invoked the transient formation of an NHC stabilised complex aluminene bound to an iron carbonyl, but isolation of this species was not possible.^70^ Instead it readily partook in the C–H activation of the THF solvent.

**Fig. 21:**
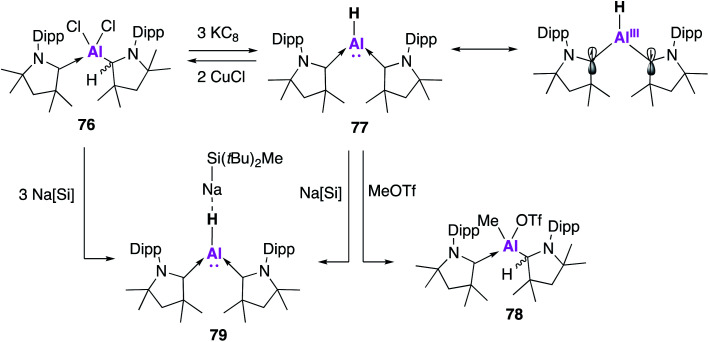
The synthesis of CAAC stabilised aluminene **77** and its reactivity.

Braunschweig and co-workers sought to exploit the exceptional σ-donating and π-accepting properties of the cyclic (alkyl)(amino) carbene (CAAC) ligand system in order to stabilise an aluminene fragment under the harsh reaction conditions required.^71^ Reduction of the alkyldichloroalane precursor (**76**) with potassium graphite (KC_8_) saw the ready formation of an aluminene species stabilised by two CAAC ligands (**77**, Fig. 21). Both the hydride and deuteride analogues could be synthesised, with the hydride transferred from the protonated CAAC ligand as confirmed by deuteration experiments. This reaction was remarkably high yielding (70%) when compared with other monomeric Al(i) syntheses. Compound **77** was found to be centrosymmetric in the solid-state, with a trigonal planar geometry at the aluminium centre with a highly electron rich hydride bound to aluminium. DFT calculations revealed that **77** was best described as an Al(i) hydride with a non-negligible contribution (approximately 1/3) from an open-shell Al(iii) diradical resonance form (Fig. 21). Despite the nucleophilicity of **77**, its reaction with a series of small molecules, including H_2_, CO_2_ and CO, led to a variety of decomposition products; this is marked contrast to most other examples of low oxidation state aluminium complexes. However, methyl triflate (MeOTf) underwent nucleophilic substitution at aluminium to form **78**. Nucleophilic attack at the methyl group is proposed to form an unstable Al(iii) cation, thus the Al-bound hydride migrates back to a CAAC centre. The reversible 2-fold oxidation of **77** was observed in the presence of CuCl, whilst reaction with Na[Si*t*BuMe_2_] formed the 1 : 1 complex **79** as determined by single crystal XRD. Interestingly, dissolution of single crystals of **79** in C_6_D_6_ revealed only resonances of free **77** and Na[Si*t*BuMe_2_], suggesting that **79** formed *via* the co-crystallisation of **77** and the sodium silane, which was likely facilitated by electrostatic interactions between the Al–H and the sodium atom.

## Conclusions and outlook

Over just the last four years a wealth of new research into the low oxidation state chemistry of aluminium has emerged. This has largely been possible through the rational design of complexes, with judicious choice of the ancillary ligands which are essential to enabling stabilisation of these highly reactive compounds. This research has included the formation of the first aluminium–aluminium double bond (**4**), isolation of several examples of alumanyl anions (**17**, **32**, **44**, **49** and **58**) and the first isolatable example of an aluminene species (**77**). These highly unusual compounds have demonstrated unique reactivity towards the activation of small molecules and have enabled the formation of both bimetallic and heterobimetallic compounds. In particular, Aldridge and Goicoechea's potassium alumanyl species has shown utility in a wide range of bond activation processes and has been used to provide access to unusual chemical structures such as the seven membered metallocycle formed through the C–C bond activation of benzene. Aluminium–aluminium doubly bonded compounds have also proven to be efficient pre-catalysts for the hydroboration of CO_2_ as well as the dehydrogenative coupling of amine boranes. This remarkable breadth of reactivity is largely driven by the ready accessibility of the frontier molecular orbitals (FMOs), with combinations of high energy HOMOs/low-lying LUMOs leading to narrow HOMO–LUMO gaps. These FMOs are able to be tuned by the employment of ligands with different electronic and steric properties, with lessons taken from other aspects of main-group and transition metal chemistry. Here, a careful balance must be struck between complex stabilisation and facilitating access to the powerful reaction potential of these compounds. Through expansion of the scope of ligands used to stabilise low oxidation state aluminium complexes we can expect to delve further into the chemical capabilities of such molecules.

That said this is a challenging pursuit. Nearly 30 years after Schnöckel reported the first stable Al(i) molecule, there remain only two types of mono-anionic ligand capable of stabilising monomeric Al(i) centres. This will continue to be a valuable synthetic target due to their diverse and well-defined reactivity; employing ligands that are not susceptible to further reaction (β-diketiminates are known to undergo deprotonation/bond activation) will be particularly important in this regard. Additionally, whilst the group of low oxidation state aluminium compounds documented herein has found utility in a large range of bond activation processes, the further reactivity and transfer of the molecular fragments formed has yet to be much explored. The development of aluminium chemistry lags much behind boron in this respect, which is widely used to deliver organic building blocks for the formation of more complex molecules across academia and chemical industry. Further challenges within the field include creating synthetic protocols that are cheap, accessible and yield high quantities of the reagent, something which has hindered the more universal use of low oxidation state aluminium as a reagent. This will also assist with establishing the more wide-spread use of such aluminium compounds as stoichiometric reducing agents. Finally, the isolation of stable Al(i) species presents obvious questions about whether aluminium can be used in redox based catalysis. Examples of reversible redox reactivity at aluminium centres provide some hope in this respect, but without the presence of additional empty orbitals or vacant coordination sites it is difficult to envisage how transition metal like redox catalysis could be achieved; thus, further complex development is undoubtably required to reach such heights. Nevertheless, with such landmark discoveries made on a yearly basis, the future of aluminium chemistry looks very bright indeed.

## Conflicts of interest

There are no conflicts of interest to declare.
